# Generation of *Trichoderma harzianum* with *pyr4* auxotrophic marker by using the CRISPR/Cas9 system

**DOI:** 10.1038/s41598-020-80186-4

**Published:** 2021-01-13

**Authors:** Amanda A. Vieira, Giovanni R. Vianna, Jessica Carrijo, Francisco J. L. Aragão, Pabline M. Vieira

**Affiliations:** 1 Programa de Pós-Graduação em Proteção de Plantas, Instituto Federal Goiano, Rodovia Geraldo Silva Nascimento, Km 2,5, Urutaí, GO CEP: 75790-000 Brazil; 2 Embrapa Recursos Genéticos e Biotecnologia, PqEB W5 Norte, Brasília, DF CEP: 70770-900 Brazil; 3 Instituto Federal de Brasília, Campus Samambaia, Samambaia Sul, Brasília, DF CEP 72320-328 Brazil

**Keywords:** Functional genomics, Biotechnology, Microbiology

## Abstract

*Trichoderma harzianum* is a filamentous fungus used as a biological control agent for agricultural pests. Genes of this microorganism have been studied, and their applications are patented for use in biofungicides and plant breeding strategies. Gene editing technologies would be of great importance for genetic characterization of this species, but have not yet been reported. This work describes mutants obtained with an auxotrophic marker in this species using the CRISPR (Clustered Regularly Interspaced Short Palindromic Repeats)/ Cas (CRISPR-associated) system. For this, sequences for a guide RNA and *Cas9* overexpression were inserted via biolistics, and the sequencing approach confirmed deletions and insertions at the *pyr4* gene. Phenotypic characterization demonstrated a reduction in the growth of mutants in the absence of uridine, as well as resistance to 5-fluorotic acid. In addition, the gene disruption did not reduce mycoparasitc activity against phytopathogens. Thus, target disruption of the *pyr4* gene in *T. harzianum* using the CRISPR/Cas9 system was demonstrated, and it was also shown that endogenous expression of the system did not interfere with the biological control activity of pathogens. This work is the first report of CRISPR Cas9-based editing in this biocontrol species, and the mutants expressing Cas9 have potential for the generation of useful technologies in agricultural biotechnology.

## Introduction

Species of the fungal genus *Trichoderma* are important biocontrol agents (BCAs) used in agriculture, and they are also industrial producers of enzymes^1–5^. Several bioformulations have been reported as both mycoparasites and nematode parasites and are already registered^3,6^. Additionally, there are a number of studies on biotechnological applications of enzymes from these organisms for biodiesel production^4,5,7,8^ and in transgenic plants leading to resistance to biotic and abiotic stresses^9,10^. Therefore, efficient molecular tools are essential for structural and functional genomics investigations in *Trichoderma* industrial and biocontrol species^6,11,12^.

Based on studies in a number of filamentous fungi, it is very difficult to achieve gene deletion in *Trichoderma* biocontrol strains using traditional genetic approaches^13–15^. They have an inefficient homologous recombination machinery and, because the fungus reproduces asexually, it prefers to perform non-homologous recombination, which results in a low frequency of correct genomic integration^16–20^. These challenges could be overcome by the CRISPR/Cas9 system, a gene editing technique in which nucleotides can be inserted, replaced or removed from the genome through endonucleases^13,19–21^. To date, work on the CRISPR/Cas9 gene editing system has occurred exclusively in *Trichoderma reesei*, and has been able to generate either selective markers or strains with increased protein production^16,20,21^. However, this species is an industrial producer of cellulases and hemicellulases that already present a high number of mutants produced using traditional genetic approaches, including strains with *pyr* as an auxotrophic marker^22–25^.

*Trichoderma harzianum* is a soil-borne fungus used in biofungicides for the biological control of agricultural diseases that affect crops of economic importance, such as soybean, rice, corn, tomato, tobacco, and bean^11,26,27^. This microorganism is cosmopolitan and performs biocontrol through several mechanisms of action, including antibiosis, competition for nutrients and mycoparasitism, in addition to promoting plant growth, inducing greater tolerance to stresses and increasing seed germination rates^2,28,29^. These beneficial effects promoted by *T. harzianum* on plants are possible due to their ability to colonize and penetrate the roots of plants and to carry out symbiotic relationships^2,3,11,12^.

Due the fact that *T. harzianum* is among the bioagents most used in today’s agriculture worldwide^11,30,31^, there is increasing interest in understanding the modes of action of this biocontrol fungus and the underlying molecular processes in greater detail. The recent development of the CRISPR/Cas9 gene editing technique could form the basis for large-scale genetic manipulations of this biocontrol fungus, but the establishment of additional selection markers is also crucial. Thus far, only a limited number of selection markers have been available for genetic transformation of *T. harzianum*, and OMP-decarboxylase deletion (*pyr* −) has proved to be a reliable auxotrophic marker for filamentous fungi^5,14,22,32,33^. Furthermore, the effects of gene deletion together with Cas9 overexpression in a biocontrol fungus is innovative. The use of the CRISPR/Cas9 gene editing system to disrupt the *pyr4* gene in *T. harzianum* represents a promising strategy for validating the technique in this fungus; it also prepares the ground for further work on gene editing and the functional analysis of this system during mycoparasitism.

## Results and discussion

Since genetic tools have scarcely been developed for most filamentous fungus, it is currently difficult to employ genetic engineering in understanding the biology of *Trichoderma spp*. and to fully exploit them industrially^8,34^. Moreover, the frequency of homologous recombination in some species is traditionally very low, time-consuming and sometimes troublesome^16,19,20,33^. For these reasons, there is a demand for developing versatile methods that can be used to genetically manipulate this biocontrol species. Therefore, gene editing technologies represent a highly promising alternative in genetic engineering of *T. harzianum* and have prompted us to establish new mutant lines for large-scale genetic manipulations. To facilitate this, we have developed a CRISPR/Cas9-based system adapted for use in this biocontrol fungus.

**Figure 1:**
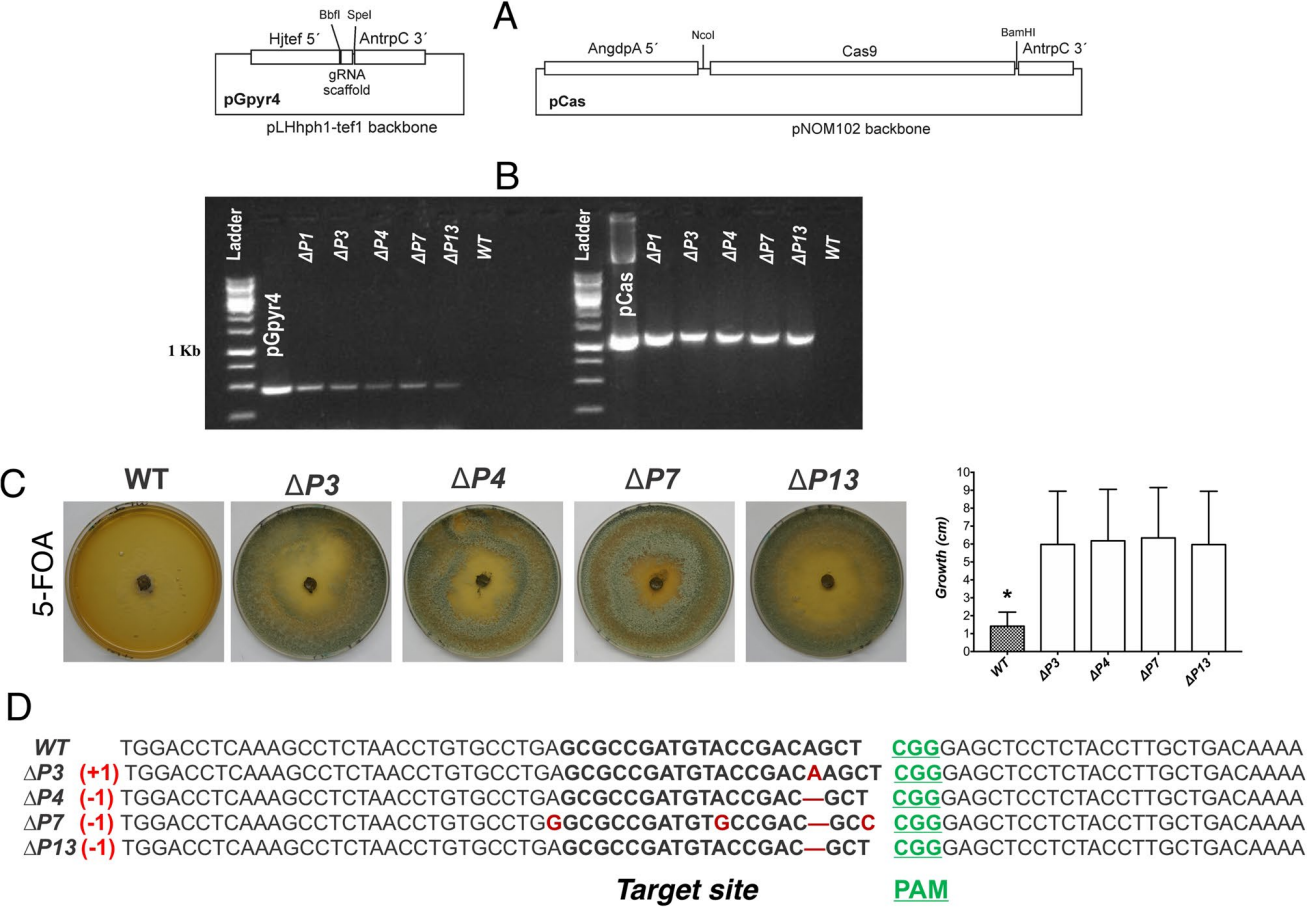
Screening of *T. harzianum* mutants resistant to 5′-FOA. (**A**) Schematic representation of vectors used to transform fungus spores. (**B**) Genomic DNA of *T. harzianum* wild-type (WT) and FOA-resistant mutants (*ΔP3*, *ΔP4*, *ΔP7*, and *ΔP13*) was isolated and screened by PCR for the presence of the *Cas9* gene which yielded a specific amplicon of 1242-bp. (**C**) Phenotype and growth of *T. harzianum* wild-type (WT) and FOA-resistant mutants (*ΔP3*, *ΔP4*, *ΔP7*, and *ΔP13*). *Bars marked with asterisk differ significantly (*P* < 0.05). (**D**) A fragment from *pyr4*, which was used for sequencing, confirmed mutants’ indels at target region. The sgRNA guiding sequence is highlighted in bold.

To CRISPR/Cas9-mediated genome editing, both the endonuclease and the sgRNA need to be present in the nucleus of the target organism^13^. In order to create vectors suitable for *pyr4* gene editing in *T. harzianum*, the respective *Cas9* sequence was inserted in pNOM102 plasmid^35^, under control of the *A. nidulans gpdA* promoter and *trpC* terminator. Subsequently, the gRNA sequence for *pyr4* was inserted in pLHhph1-tef1^36^ plasmid, containing a *hygromycin phosphotransferase* gene (*hyg*) from *E. coli* as a dominant selectable marker. The resulting plasmids, pCas and pGpyr4 (Fig. 1), were used for fungal transformation procedure.

Protoplast transformation of *Trichoderma* species includes PEG/CaCl_2_, electroporation, and *A. tumefaciens*-mediated strategies. However, preparation of protoplasts using various cell-wall degrading enzymes is time-consuming and expensive. In this way, biolistic bombardment is simple and versatile, as plasmids can be delivered into *Trichoderma* intact conidia.

Disruption of *pyr4* confers 5-fluoroorotic acid (5-FOA) resistance to *T. harzianum*. Mutants which are defective in *pyr4* are prototrophic strains resistant to 5-FOA, which is converted by orotidine-5′-monophosphate (OMP)-decarboxylase to the toxic intermediate 5-fluoro-UMP^37^. In this work, biolistic have been employed successfully for introducing *Cas9* and gRNA in *T. harzianum*, and positive transformants presented both plasmids by PCR, as described in the materials and methods section. Colonies began to appear 3 days after plating of conidia on selective medium containing 5-FOA and uridine. Fourteen transformants were generated in two bombardment experiments (12 plates), with an efficiency of between 0 and 3 co-transformants per plate. Four *T. harzianum* mutants (named *ΔP3, ΔP4, ΔP7 and ΔP13*) that showed the codon-optimized *Cas9* gene (Fig. 1B) after selection in 5-FOA medium and single spore isolation (Fig. 1C) were used for assays.

Correspondingly, sequencing approaches were used to carry out a comparative *pyr4* analysis with the wild-type strain, and indels at the gRNA target were shown for all mutants (Fig. 1D). Thus, the CRISPR/Cas9 technique enabled the production of *T. harzianum* strains with an auxotrophic marker that also expressed the *Cas9* gene. From a practical perspective, our work introduces a powerful genome-editing approach in mitotically stable mutants with endogenous *pyr*4 gene disruption accomplished by Cas9 expression. This system could be versatile and simple, as new mutagenesis can be achieved in *T. harzianum* lines by re-transforming with a single plasmid containing RNA guide.

**Figure 2:**
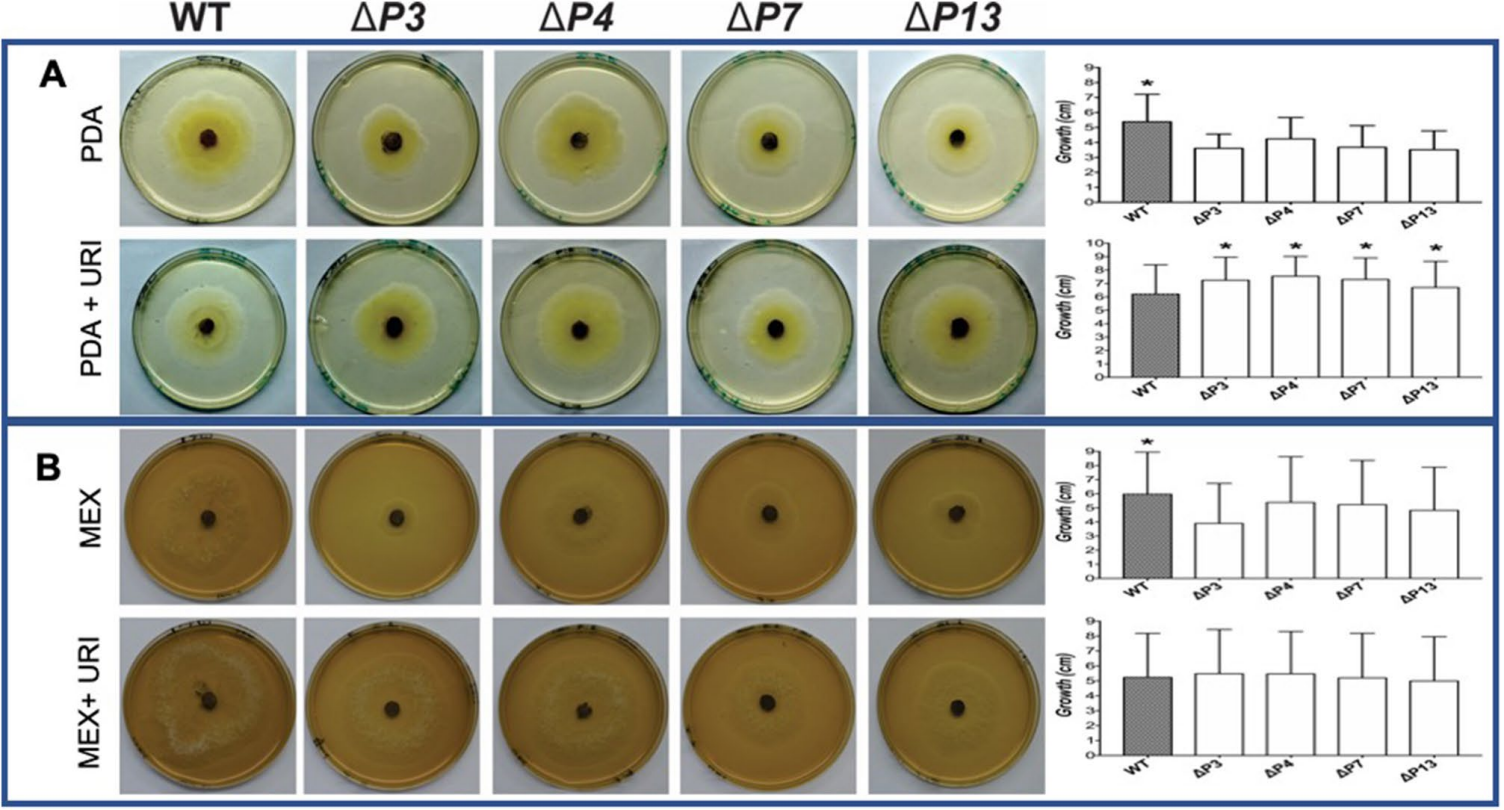
Phenotype analysis of *T. harzianum* mutants for uridine auxotrophy. *T. harzianum* wild-type and mutants (*ΔP3*, *ΔP4*, *ΔP7*, and *ΔP13*) were grown at 28 °C in absence or presence of uridine. Pictures after 2 days from bioassays conducted in PDA (**A**) and MEX (**B**) media. *Bars marked with asterisk differ significantly (*P* < 0.05).

One of the most important advancements in recent years, for improving the performance of research with *Trichoderma* species is the development of auxotrophic strains. Disruption of *pyr4* also generates auxotrophic strains defective for uridine (uracil). In our work, we present the successful establishment of this selection marker for the genetic transformation of the biocontrol fungus *T. harzianum*. Indeed, results from assays in PDA medium without uridine demonstrated that mutants (*ΔP3, ΔP4, ΔP7 and ΔP13*) presented lower growth ratios compared to the wild-type (WT) strain (Fig. 2A). Moreover, assays in PDA with uridine revealed that all mutants showed higher growth ratios compared to WT (Fig. 2A). In relation to assays conducted in MEX medium, it was demonstrated that *pyr4* disruption also reduced the mutants’ growth ratio in the absence of uridine. However, addition of uridine to MEX medium only reestablished mutants’ growth ratio similarly to WT (Fig. 2B). In this way, the CRISPR/Cas9 system caused insertions and deletions (indels) in target regions of the *pyr4* gene and successfully interrupted gene function. In addition, phenotypic analyses confirmed that these mutants need a complement of uridine in the medium to present similar growth to the wild-type, thus certifying the presence of this convenient selection marker.

Microorganisms with *pyr*-negative marker are widely used in biotechnological processes, industries and research^14,21,25^. Indeed, genomic research with the industrial species *T. reesei* entered a new era after *pyr*-negative strains became available. Nevertheless, *T. reesei* with this auxotrophic marker has been produced using both traditional genetic approaches^22,25^ and, recently, CRISPR/Cas9 technology^20^. Additionally, research using the CRISPR/Cas9 system reveals that these markers are exclusive to *T. reesei*^15,16,20,38^. These studies used protoplast or Agrobacterium transformation methods and described only in vitro transcription of gRNA^15,16,20,38^. Our work was successfully carried out by biolistic direct transformation of *T. harzianum* with the Cas9/gRNA complex, and it may be an alternative means to achieve fast gene disruption, while the overexpression of a codon-optimized *Cas9* provides a means to speed up genome editing in this biocontrol fungus. In addition, the use of Cas9 and gRNA in separate plasmids allows the generation of new edition vectors by manipulating only the gRNA vector in a simple and cheaper manner. Mutants overexpressing Cas9 could be re-transformed with new gRNA vectors, taking advantage of the *pyr4* auxotrophic marker. Despite these advantages, there has been no report of using such a technique in other *Trichoderma* biocontrol species.

*Trichoderma harzianum* is a cosmopolitan filamentous fungus that displays a remarkable range of applications in agricultural biotechnology^2,3^. Because of its ability to antagonize plant–pathogens as well as stimulating plant growth and defense responses, some strains are used in bioformulation for biological control^2,11,26,27^. In this way, gene disruption has been a critical technique for improvement of *T. harzianum* strains and biocontrol studies.

**Figure 3:**
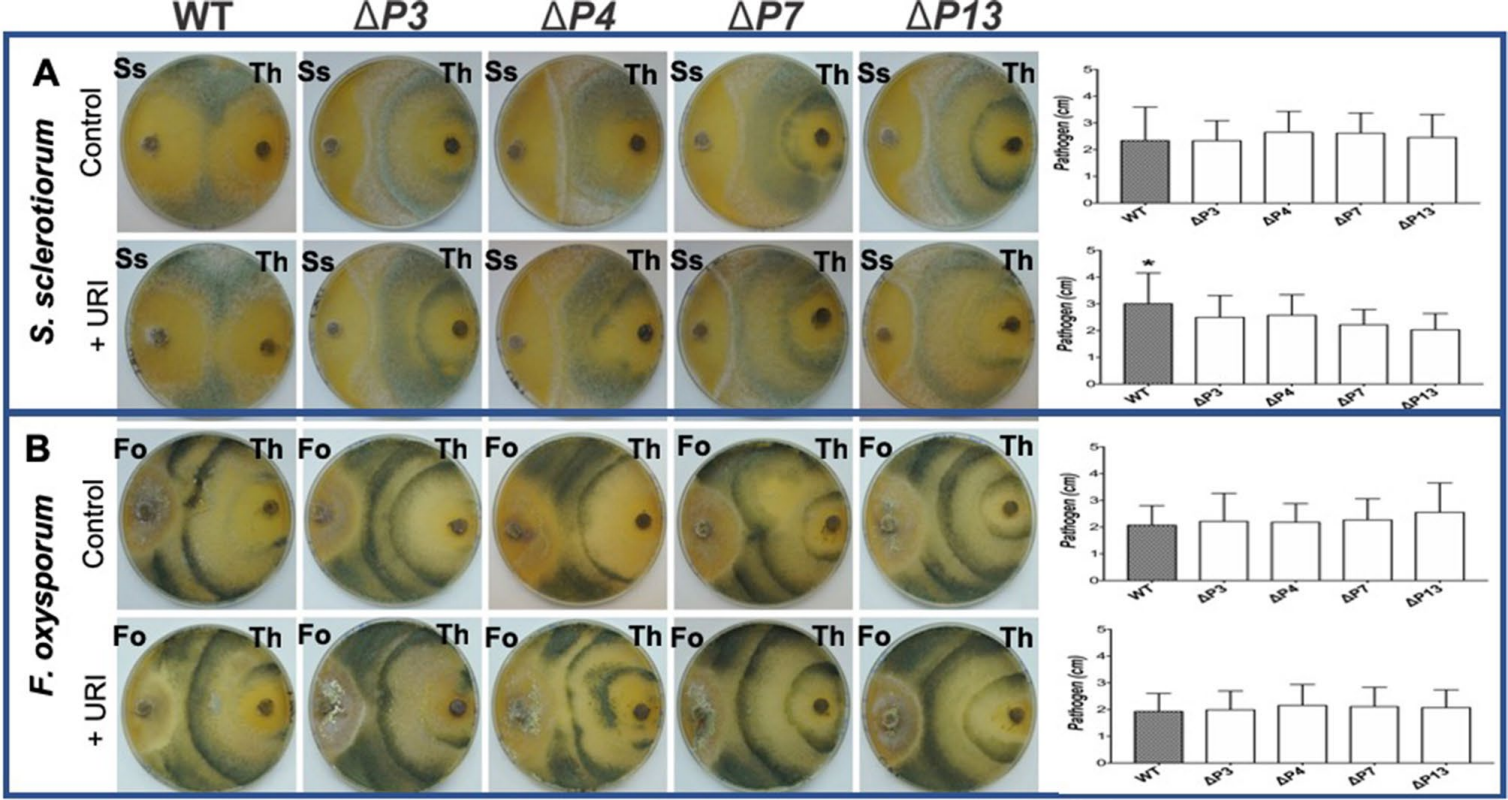
Mycoparasitic abilities of uridine auxotrophic mutants. The antagonistic activity of mutants (*ΔP3*, *ΔP4*, *ΔP7*, and *ΔP13*) in comparison to the *T. harzianum* wild-type was assessed in plate confrontation assays using *Sclerotinia sclerotiorum* (**A**) or *Fusarium oxysporum* (**B**) as host fungus. Bioassays were conducted in presence and absence of uridine. *Bars marked with asterisk differ significantly (*P* < 0.05).

The effects of *pyr4* disruption and *Cas9* overexpression on the mycoparasitic interaction between *T. harzianum* and fungal hosts were assessed in plate confrontation assays. In relation to *S. sclerotiorum* assays, we observed that the absence of uridine did not affect mutants’ ability to mycoparasitize this pathogen, compared to the WT strain (Fig. 3A). However, confrontation assays carried out in the presence of uridine demonstrated that mutants decreased *S. sclerotiorum* overgrowth compared to wild-type (Fig. 3A).

Confrontation assays were also performed to compare mycoparasitic abilities of *T. harzianum* strains against *F. oxysporum* either in absence or presence of uridine (Fig. 3B). No differences between the tested strains were observed for the inhibition of *F. oxysporum* in all media analyzed (Fig. 3B). Bioassays with pathogens demonstrated that *pyr4* gene disruption (OMP-decarboxylase), important for the pyrimidine synthesis pathway, in addition to Cas9 expression, did not reduce the mycoparasitic activity of mutants. Thus, our results underscore the use of the CRISPR/Cas9 system in *T. harzianum* has many prospects for functional analysis of biocontrol genes, metabolic modifications, and the selection or production of new strains for biotechnological uses.

## Conclusion

For the first time, this work successfully established a promising approach for genome editing in the biocontrol fungus *T. harzianum*. Mutants produced with an auxotrophic marker and *Cas9* overexpression provide a tool for functional analysis of biocontrol genes, selection of strains for bioformulations, and the generation of new strains for biotechnological uses.

## Materials and methods

### Microorganisms and culture conditions

*Trichoderma harzianum* ALL42 (Enzymology group collection—UFG/ICB) was used for this study. *Fusarium oxysporum* and *Sclerotinia sclerotiorum* were from the EMBRAPA–CNPAF culture collection. The microorganisms were maintained on potato/dextrose/agar (PDA) plates with periodic sampling and stored at 4 °C in EMBRAPA/CNPAF before use.

### Construction of the CRISPR/Cas9 gene edition system

For the construction of the CRISPR/Cas9 editing system, the *Cas9* gene coding sequence from *S. pyogenes* was codon-optimized for expression in *Trichoderma harzianum* and synthesized by Epoch Life Science, Inc. (Sugar Land, TX, USA) (Supplementary Material). The *Cas9* sequence was inserted in the pNOM102 plasmid^35^ between the constitutive *A. nidulans gpdA* promoter (GenBank accession number: Z32524.1, position 61 to 2129) and *trpC* terminator (GenBank accession number: X02390.1, position 3466 to 4168), generating the vector pCas. The gRNA sequence for *pyr4* (JGI ID: 480432, Fig. 1) edition was designed using the online E-CRISPR design server (http://www.e-crisp.org/E-CRISP/) and inserted downstream of the constitutive *H. jecorina* (*T. reesei*) *tef1* promoter of the plasmid pLHhph1-tef1^36^, generating the vector pGpyr4. The two final vectors (pCas and pGpyr4; Fig. 1) were used for biolistic transformation in a 1:1 molar ratio.

### Preparation of microparticles and cells for bombardment, and biolistic co-transformation of *T. harzianum*

Transformation procedure was based on previous protocols^18,39,40^, with some essential modifications described in the following. DNA was bound to 0.2-μ-diameter tungsten particles (M5, Sylvania Inc.) by mixing sequentially in a microcentrifuge tube: 50 μl microparticles (60 mg ml^−1^ in 50% glycerol), 5 μl (1 μg μl ^−1^) of each plasmid constructed (pCas and pGpyr4), 50 μl CaCl_2_ (2.5 M) and 20 μl spermidine free-base (100 mM). After 10 min incubation, the DNA-coated microparticles were centrifuged (15,000×*g*, 10 s) and the supernatant removed. The pellet was washed with 150 μl 70% ethanol and then with absolute ethanol. The final pellet was resuspended in 24 μl of absolute ethanol and sonicated for 2 s, just before use. Aliquots of 3 μl were spread onto carrier membranes (Kapton, 2 mil, DuPont) which were allowed to evaporate in a desiccator at 12% relative humidity.

The target material for transformation by microparticle bombardment was *Trichoderma harzianum* (ALL42) intact conidia. A suspension of conidia, previously produced by cultivation of the fungus on potato-dextrose agar was prepared by harvesting the conidia from the plate, suspending them in 0.9 M NaCl, and separating them from mycelial carryover by filtration through a column filled with glasswool. A conidial suspension (30 μl) containing 1.7 × 10^7^ spores ml^−1^ was bombarded with the DNA-coated microparticles utilizing a high pressure helium-driven particle acceleration device built in our laboratory^40^. The relative humidity in the biolistic laboratory was 50%, the gap distance from shock wave generator to the carrier membrane was 8 mm, the carrier membrane flying distance to the stopping screen was 13 ram, the DNA-coated microparticles flying distance to the target was 80 mm, the vacuum in the chamber was 27 inches of Hg and the helium pressure utilized in all experiments was 1 200 psi. After the bombardment, transformants were incubated at 28 °C on yeast extract/agar (MEX) plates containing 5-FOA (1.5 g/L; Fermentas, St. Leon-Rot, Germany) and uridine (10 mM).

### Selection and stabilization of co-transformants

Inoculated plates were incubated at 28 °C for up to 10 days during which plates were periodically examined directly for *Trichoderma harzianum* conidia development. Colonies appearing after incubation were picked using a sterile needle and transferred to fresh selective medium. Mutants were sub-cultured for a further three cycles of mycelial growth and conidiation.

### Molecular analysis of *T. harzianum* mutants and sequencing

Following three rounds of single-spore isolation, we obtained 14 mutants by phenotypic analysis (5′FOA resistance). Genomic DNA from four co-transformed strains were isolated as described previously^22^ and screened by PCR amplification with primers specific for pCas cassette (Cas9_RNAgCheC: 5′-CTGCAAGGCGATTAAGTTGG-3′/ Cas9_3897F: 5′-ACAGCATAAGCACTACCTCG-3′) and also pGpyr4 vector (hygF:5′-CACGTTGCAAGACCTGCCTGAA-3′/ hygR:5′-TCCGGATGCCTCCGCTCGAAGTA-3′). The amplification conditions were: an initial denaturation step of 2 min at 94 °C, followed by 30 cycles of 30 s at 94 °C, 30 s at 55 °C and 60 s at 72 °C, and a final extension step of 10 min at 72 °C. The *pyr4* gene fragment, which was used for sequencing and further analysis, was amplified (pyrF: 5′-AGCTCTAACCTGTGCCTGA-3′/ pyrR: 5′-AAGGTAGAGGAGCTCCCG-3′), cloned into the pGEMT-Easy vector according to standard procedures and sequenced using SP6 universal primer. DNA from the wild type (WT) strain was included as control.

### Growth and direct confrontation assays

To analyze *Trichoderma harzianum* mutants for uridine auxotrophy, mycelium-covered plugs were placed at the center of fresh PDA or MEX plates supplemented with 10 mM uridine and incubated at 28 °C for 7 days. Antagonism activity of *T. harzianum* WT and mutants against pathogens was performed as a plate confrontation assay as described previously^41^, and colony diameter measurement was taken for a period of 7 days. Two pathogens (*Sclerotinia sclerotiorum* and *Fusarium oxysporum*) were independently evaluated during confrontation with *T. harzianum* strains in presence and absence of uridine. All experiments were performed using three biological replicates.

### Statistical analysis

The data were analyzed for normality (Shapiro–Wilk’s tests) and for homogeneity (Bartlett’s tests). Data that were not normal were transformed using (x + 0.5)^1/2^. Afterwards, data were subjected to ANOVA, and means were separated by Dunnett’s test at 5% probability whenever ANOVA was significant. The statistical analysis was performed using software R, version 3.2.2 (R Core Team, 2016), and graphical work was carried out using GraphPad Prism version 7.0 software (La Jolla, CA, USA).

## Supplementary Information

Supplementary Information
