# Supplementary material for: Generation of Trichoderma harzianum with pyr4 auxotrophic marker by using the CRISPR/Cas9 system
Source: Sci Rep. 2021 Jan 13;11:1085. doi: 10.1038/s41598-020-80186-4 (PMC7806921; doi:10.1038/s41598-020-80186-4)
Supplement: Supplementary file 1 — Supplementary Information [file 41598_2020_80186_MOESM1_ESM.docx]

**Supplementary Information**

**Generation of *Trichoderma harzianum* with *pyr4* auxotrophic marker by using the CRISPR/Cas9 system**

Amanda A. Vieira^1^; Giovanni R. Vianna ^2^; Jessica Carrijo^2^; Francisco J. L. Aragão^2^; Pabline M. Vieira ^1,2,3∗^

^1^ Programa de Pós-graduação em Proteção de Plantas, Instituto Federal Goiano, Rodovia Geraldo Silva Nascimento, km 2,5. CEP: 75790-000. Urutaí, GO, Brasil.

^2^ Embrapa Recursos Genéticos e Biotecnologia, PqEB W5 Norte, CEP: 70770-900, Brasília, DF, Brazil.

^3^ Instituto Federal de Brasília, *Campus* Samambaia, Samambaia Sul, CEP 72320-328. Brasília, DF, Brazil.

∗ *Corresponding author:*

Instituto Federal de Brasília, *Campus* Samambaia, Samambaia Sul, CEP 72320-328. Brasília, DF, Brazil.

**Sequence of the optimized Cas9 gene coding sequence.**

ATGGACTATAAGGATCACGATGGAGACTACAAGGATCATGATATTGACTATAAGGACGATGATGATAAGATGGCTCCCAAGAAGAAGCGCAAGGTGGGTATTCATGGGGTTCCAGCTGCCGACAAGAAGTATAGCATTGGTCTTGATATCGGAACCAATTCAGTCGGTTGGGCTGTGATCACCGACGAATATAAGGTTCCTTCAAAGAAGTTCAAGGTTTTGGGTAACACGGATCGTCATAGCATCAAGAAGAATCTTATTGGTGCCCTTCTGTTCGACTCAGGCGAGACTGCAGAGGCCACTCGATTAAAGAGGACTGCTAGGCGTCGATATACACGTAGGAAGAACAGGATTTGCTATCTTCAAGAGATCTTCTCTAATGAAATGGCAAAGGTGGACGATTCCTTTTTCCACAGGCTGGAAGAGAGCTTCTTGGTTGAGGAAGATAAGAAGCACGAACGTCACCCTATTTTTGGAAATATCGTGGATGAAGTCGCTTACCACGAGAAGTATCCAACTATCTACCATCTCAGGAAGAAGTTGGTGGACAGCACTGACAAGGCAGATCTCAGGCTCATCTATCTCGCCCTGGCTCATATGATTAAGTTTAGGGGTCACTTCCTCATTGAAGGCGATCTCAACCCCGATAACTCCGACGTTGATAAGTTATTCATCCAGCTTGTTCAAACCTACAATCAACTGTTCGAAGAGAACCCTATTAACGCGTCTGGGGTGGATGCAAAGGCAATTCTCTCTGCCAGGCTTTCTAAGTCTAGGCGTTTGGAGAATCTTATTGCACAGTTGCCCGGAGAAAAGAAGAATGGATTGTTCGGGAACTTGATCGCTTTAAGCCTCGGACTGACCCCAAACTTCAAGTCCAATTTCGACTTGGCAGAAGACGCAAAGTTGCAGCTCAGCAAGGACACATACGACGATGACCTGGACAATCTTTTAGCACAAATTGGAGATCAGTACGCAGATCTCTTCTTAGCTGCCAAGAACTTATCTGATGCTATCTTGCTCAGCGACATTTTGCGAGTGAATACAGAGATCACAAAGGCACCACTCTCCGCTTCCATGATCAAGCGGTATGACGAGCATCACCAAGATCTCACTCTGCTTAAGGCACTGGTCAGGCAACAGCTTCCAGAAAAGTACAAGGAGATCTTCTTTGATCAGTCGAAGAATGGCTACGCCGGTTATATCGATGGAGGCGCTTCTCAGGAGGAATTTTACAAGTTTATTAAGCCAATCCTCGAGAAGATGGACGGCACTGAGGAATTGCTGGTTAAGCTCAATCGCGAAGATTTGTTAAGGAAGCAGAGGACTTTCGATAATGGGTCTATTCCTCATCAGATCCATCTCGGAGAGTTGCATGCAATCTTGAGGAGGCAGGAAGATTTTTATCCTTTCTTGAAGGATAATCGGGAAAAGATCGAGAAGATTCTGACCTTTAGGATTCCTTACTATGTCGGACCTCTGGCAAGGGGTAACTCTAGGTTCGCTTGGATGACTAGGAAGTCTGAAGAGACAATTACGCCTTGGAACTTTGAAGAGGTGGTCGATAAGGGAGCGTCTGCTCAGAGCTTCATCGAAAGGATGACTAATTTCGATAAGAATCTCCCTAATGAAAAGGTGCTTCCTAAGCACTCACTCCTCTATGAGTACTTTACAGTGTACAATGAACTGACCAAGGTTAAGTACGTTACCGAAGGGATGAGGAAGCCTGCTTTTTTGTCTGGTGAGCAAAAGAAGGCCATTGTGGACCTTCTGTTTAAGACAAACCGAAAGGTGACTGTCAAGCAACTTAAGGAGGATTACTTTAAGAAGATTGAATGCTTTGATTCTGTCGAGATTTCAGGGGTCGAGGACCGCTTCAACGCCAGCTTGGGCACCTATCATGATCTCCTTAAGATTATCAAGGATAAGGATTTTCTGGATAACGAAGAGAACGAAGACATTTTAGAGGACATCGTTCTTACTCTGACTCTTTTTGAAGATCGCGAAATGATCGAGGAAAGGCTGAAGACTTATGCACATTTGTTTGATGACAAGGTGATGAAGCAACTTAAGAGGCGAAGGTACACAGGGTGGGGTCGACTCTCAAGGAAGCTCATTAACGGTATCCGTGACAAGCAAAGCGGAAAGACTATTCTTGATTTTCTTAAGAGCGACGGATTCGCCAACCGAAATTTCATGCAGCTTATCCACGATGACAGCCTTACCTTTAAGGAAGATATTCAAAAGGCACAAGTCTCGGGCCAAGGTGACTCCTTGCACGAGCATATTGCGAATTTAGCTGGAAGCCCAGCTATCAAGAAGGGGATTTTGCAAACCGTTAAGGTCGTTGATGAATTGGTCAAGGTTATGGGACGTCATAAGCCAGAGAACATCGTTATCGAAATGGCTAGGGAAAACCAGACAACCCAGAAGGGCCAAAAGAACTCGAGGGAACGGATGAAGAGGATTGAAGAGGGTATTAAGGAGTTGGGTTCACAAATTCTTAAGGAGCATCCCGTCGAAAACACCCAGCTCCAGAATGAGAAGTTATACCTCTACTATCTTCAGAACGGACGCGATATGTATGTTGATCAAGAGCTGGATATTAACAGGCTCAGCGATTACGATGTTGATCATATTGTGCCTCAATCCTTCCTCAAGGATGACAGCATTGATAACAAGGTCCTGACTAGGAGCGACAAGAACCGCGGCAAGTCTGATAACGTCCCGTCCGAGGAAGTGGTCAAGAAGATGAAGAATTATTGGCGCCAACTCCTCAATGCAAAGCTCATTACTCAGCGTAAGTTCGATAACTTGACTAAGGCTGAACGCGGAGGTCTGTCGGAACTCGATAAGGCCGGTTTTATTAAGAGGCAGTTGGTGGAGACCCGTCAAATTACTAAGCACGTTGCTCAAATCCTGGACTCAAGGATGAATACAAAGTATGATGAAAATGACAAGCTCATTAGGGAGGTTAAGGTTATCACATTAAAGTCAAAGCTCGTGAGCGACTTCCGCAAGGATTTTCAATTTTATAAGGTTAGGGAGATCAACAATTATCATCACGCCCACGACGCCTACTTGAATGCTGTTGTGGGGACAGCCTTGATCAAGAAGTACCCGAAGTTGGAATCAGAATTTGTCTATGGCGATTATAAGGTGTACGATGTGAGGAAGATGATCGCCAAGTCCGAACAAGAAATCGGCAAGGCGACCGCGAAGTACTTTTTCTATTCAAATATCATGAATTTTTTCAAGACGGAGATCACGTTGGCTAATGGGGAGATCAGGAAGAGGCCACTTATTGAAACAAATGGCGAAACCGGTGAGATCGTCTGGGATAAGGGGAGGGACTTTGCTACAGTTAGGAAGGTTCTCTCTATGCCACAAGTCAACATTGTCAAGAAGACAGAGGTGCAGACCGGGGGTTTTTCCAAGGAGTCAATTCTTCCAAAGCGTAACAGCGATAAGCTCATCGCCAGGAAGAAGGACTGGGACCCCAAGAAGTATGGTGGATTCGACTCGCCTACGGTGGCCTACAGCGTTTTGGTTGTCGCGAAGGTTGAGAAGGGAAAGTCAAAGAAGCTCAAGAGCGTGAAGGAGTTGTTAGGAATTACTATCATGGAACGGTCTTCCTTTGAGAAGAACCCAATCGACTTTTTGGAAGCTAAGGGATATAAGGAAGTTAAGAAGGATCTTATTATCAAGCTCCCAAAGTATTCACTTTTCGAACTTGAGAACGGACGTAAGAGGATGCTTGCTTCCGCCGGAGAGTTGCAGAAGGGTAACGAGTTGGCATTACCTTCAAAGTACGTCAACTTTCTTTATTTGGCTAGCCACTATGAAAAGCTCAAGGGGAGCCCCGAGGATAATGAACAAAAGCAGCTGTTTGTCGAACAGCATAAGCACTACCTCGATGAAATTATCGAACAGATTTCTGAGTTCTCTAAGAGGGTTATCCTCGCAGATGCTAATTTGGACAAGGTGCTTAGCGCATACAACAAGCACAGGGACAAGCCTATCCGGGAGCAAGCTGAGAACATCATTCATCTTTTTACACTCACAAATCTTGGTGCGCCGGCCGCTTTTAAGTACTTCGATACCACAATCGACCGCAAGAGGTACACTTCAACTAAGGAGGTCCTGGATGCTACCTTAATCCATCAAAGCATCACGGGCCTCTATGAGACCAGGATTGATCTCTCCCAGCTCGGCGGTGATAAGAGGCCCGCAGCTACAAAGAAGGCAGGACAAGCAAAGAAGAAGAAGTGAGTTAA
